# CircNDST1 promotes papillary thyroid cancer progression via its interaction with CSNK2A1 to activate the PI3K–Akt pathway and epithelial–mesenchymal transition

**DOI:** 10.1007/s40618-022-01928-x

**Published:** 2022-10-28

**Authors:** C. Shu, S. Wang, J. Hu, M. Xu, H. Deng, Y. Maimaiti, T. Huang

**Affiliations:** 1 Department of Breast and Thyroid Surgery, Union Hospital, Tongji Medical College, Huazhong University of Science and Technology, Wuhan, China; 2 Department of General Surgery, People’s Hospital of Xinjiang Uygur Autonomous Region, Urumqi, China; 3 Department of Breast and Thyroid Surgery, Renmin Hospital of Wuhan University, Wuhan, China

**Keywords:** circNDST1, Thyroid cancer, PI3K–Akt pathway, EMT

## Abstract

**Background:**

Multiple studies have established a strong relationship between circRNA and cancer progression. Cervical lymph node metastasis is a key factor influencing the surgical approach and distant metastasis of papillary thyroid cancer (PTC). However, the role of circNDST1 in PTC has not been investigated. Our research focused on revealing the function and mechanism of action of circNDST1 in PTC.

**Methods:**

High-throughput sequencing and qPCR were used to assess the expression of circRNA in PTC tissues with extensive cervical lymph node metastasis and circNDST1 in cell lines, respectively. The proliferative effects of circNDST1 in vitro and in vivo were analyzed using CCK8, clone formation assay, EdU, and nude mouse tumorigenesis assay. The transwell scratch assay was employed in the scrutiny of the effect of circNDST1 on the migration and invasion abilities of thyroid cancer cells, while circNDST1’s influence on the PI3K–Akt pathway and the Epithelial–Mesenchymal Transition (EMT) key protein expression was evaluated utilizing RNA sequencing and western blot. RNA pull-down and RIP were used to examine the binding of circNDST1 to CSNK2A1.

**Results:**

CircNDST1 was highly expressed in PTC cell lines, but knocking it down inhibited the proliferation, migration, and invasive abilities of TPC1 and KTC1 cell lines. CircNDST1 bonded with CSNK2A1 and promoted the interaction between CSNK2A1 and Akt, leading to the activation of the PI3K–Akt pathway and EMT.

**Conclusion:**

CircNDST1’s high expression boosted thyroid cancer progression through the activation of the PI3K–Akt pathway and EMT in a CSNK2A1-dependent manner

**Supplementary Information:**

The online version contains supplementary material available at 10.1007/s40618-022-01928-x.

## Introduction

About 85% of thyroid cancer cases are papillary thyroid cancers (PTCs), which are predisposed to metastasize to lymph nodes in the neck, increasing the likelihood of recurrence and adversely affecting prognosis [1]. The metastasis of lymph nodes in the neck is a critical indicator of PTC progression, directly determining the surgical approach and the formulation of a postoperative comprehensive treatment plan [2]. Therefore, examining the internal driving forces of cervical lymph node metastasis in PTC is fundamental to finding a suitable solution.

circRNA is a non-coding RNA that forms a loop structure by covalently closing the 5´ and 3´ ends of the precursor RNA, most of which is localized in the cytoplasm, and its highly conserved and non-easily degradable structure by RNA exonucleases makes its expression stable [3]. The dysregulation of circRNA affects cell proliferation, apoptosis, epithelial–mesenchymal transition (EMT), invasion, tumor immune evasion, gene mutation, and modification, ultimately promoting tumor progression and invasive metastasis, thus becoming a potential biological marker for tumors [4].

Tyrosine kinase II (CSNK2) is a serine/threonine kinase that phosphorylates hundreds of substrates. CSNK2 consists of two catalytic subunits (CK2α and CK2α´) encoded by CSNK2A1 and CSNK2A2, respectively [5], and two regulatory subunits. Reportedly, CSNK2A1 is expressed at abnormally high levels and kinase activity in a variety of cancer cells [6–10], and it stimulates tumor proliferation, DNA damage repair, EMT, drug resistance, and other biological behaviors by phosphorylating key molecules in various signaling pathways [11]. CSNK2A1 has also been found to regulate Akt activity by phosphorylating Akt, leading to the hyperactivation of the PI3K/Akt signaling pathway [12].

In this study, we used high-throughput sequencing and identified a total of 17 up-regulated circRNAs in extensive lymph node metastatic PTC compared to tissues without extensive lymph node metastasis and then selected circNDST1 as the key subject of our investigation. We established the key role of circNDST1 in enhancing the proliferation, migration, and invasion of PTC cell lines in vitro and in vivo. Notably, we provided evidence that circNDST1 binds to and promotes the function of CSNK2A1. CircNDST1 stimulates Akt signaling pathway activation and EMT by relying on CSNK2A1’s mediation.

## Methods

### Human papillary thyroid cancer tissue specimens

PTC tissue specimens for this study were provided by patients who had their PTC surgically resected at the Union Hospital of Tongji Medical College. Cancerous tissue specimens were collected from six patients with thyroid cancer. Clinical information on the patients is shown in the attached table. Of the six patients, three had extensive lymph node metastasis, and the other three had no extensive lymph node metastasis. Tissue specimens were immediately stored in liquid nitrogen until use. Written informed consent was obtained from all patients, and the investigation was approved by the Ethics Committee of the Union Hospital of Tongji Medical College.

### RNA sequencing of circRNA extracted from human papillary thyroid cancer tissue

RNA was extracted from 3 cases with extensive metastasis in the neck lymph nodes and 3 cases without extensive lymph node metastasis in the thyroid glands. A de-ribose-specific library was then created and sequenced utilizing the Illumina HiSeq2500 platform (Illumina, San Diego, USA) according to the manufacturer’s protocol.

### Cell lines and culture conditions

Three PTC cell lines, TPC1, KTC1, and BCPAP, and normal thyroid epithelial cells, Nthy-ori3-1, were purchased from ATCC. TPC1 cells were cultured in a DMEM medium (Gibco, Carlsbad, CA, USA), while KTC1, BCPAP, and Nthy-ori3-1 were cultured in an RPMI1640 medium (Gibco, Carlsbad, CA, USA), both of which contained 10% fetal bovine serum (Gibco, Carlsbad, CA, USA). The cells were maintained in a humidified incubator at 37 °C and 5% CO_2_.

### Fluorescence in situ hybridization (FISH)

Cy3-labeled circNDST1 FISH probes designed by Guangzhou RiboBio were used to determine the localization of circNDST1 in cells. FISH was performed with a fluorescent in situ hybridization kit (RiboBio, China, cat. NO: C10910) according to the manufacturer's protocol. Nuclei were stained with 4,6-diamidino-2-phenylindole (DAPI, Beyotime, China, cat. NO: C1005), and photographs were taken with a fluorescence microscope.

### RNase R treatment

RNase R treatment was carried out according to the manufacturer’s protocol. 10 μg of total RNA was incubated with or without 10 U/μl RNase R (BioVision, USA, cat. NO: M1228) for 15 min at 37 °C, treated at 70 °C for 10 min to inactivate the Rnase enzyme, and the contents of circNDST1 and NDST1 in the RNA samples were evaluated using RT-qPCR.

### Actinomycin D assay

TPC1 cells were treated with actinomycin D (Selleck, USA, cat. NO: S8964-01) for 0 h, 4 h, 8 h, and 12 h before RNA extraction. Actinomycin D's working concentration was 5 μg/ml. The contents of circNDST1 and NDST1 in the RNA samples were assessed using RT-qPCR.

### Cytoplasmic and nuclear RNA fractionation

Nuclear and Cytoplasmic Extraction Reagents (ThermoFisher, CA, USA, cat. NO: AM1921) were used for nuclear–cytoplasm separation before RNA extraction for RT-PCR. GAPDH and U6 were employed as the respective positive controls for cytoplasmic and nuclear RNAs.

### Sanger sequencing

The amplification products of circRNA were inserted into a T-vector for Sanger sequencing by Sangon (Shanghai, China). A primer (Tsingke, Nanjing, China) was designed to confirm the back-splice junction of circNDST1.

### RNA extraction and real-time fluorescence quantitative PCR

RNA was extracted from tissues or cells using a Trizol reagent (Vazyme, Nanjing, China, cat. NO: R401-01) and reverse transcribed to cDNA per the protocol of the PrimeScript RT Reagent Kit (Vazyme, Nanjing, China, cat. NO: R323-01). Real-time quantitative PCR was used to determine the expression of target molecules. PCR primer sequences were synthesized by Tsingke (Nanjing, China) and are listed below. RNA expression fold changes were determined utilizing the 2^−ΔΔCt^ method.

GAPDH Forward Primer: 5´AGAAGGCTGGGGCTCATTTG 3´

Reverse Primer: 5´AGGGGCCATCCACAGTCTTC 3´

CircNDST1 Forward Primer: 5´ CCGCTCTGGCAGGTTCT 3´

Reverse Primer: 5´ GTTGGACAGGTGCGTCAT 3´

NDST1 Forward Primer: 5´ TTTGTTGGTCAGTGGACGATT 3´

Reverse Primer: 5´ CAGAAGATGAACAGCAGGAAAA 3´

U6 Forward Primer: 5´CTCGCTTCGGCAGCACA 3´

Reverse Primer: 5´AACGCTTCACGAATTTGCGT 3´

18S Forward Primer: 5´GTAACCCGTTGAACCCCATT 3´

Reverse Primer:5´ CCATCCAATCGGTAGTAGCG 3´

CSNK2A1 Forward Primer: 5´GAACGCTTTGTCCACAGTGA 3´

Reverse Primer: 5´TATCGCAGCAGTTTGTCCAG 3´

### DNA extraction from cells

DNA was extracted from cells following the protocol of the TIANamp Genomic DNA Extraction Kit (cat. NO: DP304-03) and used for subsequent RT-qPCR assays.

### Western blot analysis

Cells were washed with PBS, lysed in a RIPA protein extraction lysis buffer (Biosharp) containing protease and phosphatase inhibitor cocktails (MedChemExpress), and their total protein concentrations were determined with a spectrophotometer at 562 nm via the BCA assay (Vazyme, Nanjing, China). The supernatants from cell lysates were run on 10% or 12.5% acrylamide gels using SDS-PAGE and then transferred to NC membranes (Millipore). The membrane contents were blocked with 5% skim milk in Tris-buffered saline and Tween 20 (TBST) at room temperature for 1 h and incubated with antibodies at 4 ℃ overnight. Antibodies against β-actin (1: 1000, Cell signaling Technology, cat. NO: 8457 s), E-cadherin (1:1000, ABclonal, cat. NO: A3044), N-cadherin (1:1000, Proteintech, cat. NO: 22018-1-AP), Vimentin (1:1000, Proteintech, cat. NO: 10366-1-AP), Snail (1:1000, ABclonal, cat. NO: A11794), Akt (1:1000, Cell signaling Technology, cat. NO: 4691 s), p-Akt (Ser473) (1:1000, Cell signaling Technology, cat. NO: 4060 s), phosphatase and tensin homolog (PTEN) (1:1000, Proteintech, cat. NO: 22034-1-AP), p-PTEN (Thr382/383, Proteintech, cat. NO: 29246-1-AP), p70 S6K1 (ABclonal, cat. NO: A2190), p-p70 S6K1 (Thr389, ABclonal, cat. NO: AP0564), CCND1 (1:1000, Cell signaling Technology, cat. NO: 2922 s), CSNK2A1(1:1000, Proteintech, cat. NO: 10992-1-AP), and an HRP-conjugated secondary antibody (1:3000) were employed for western blotting. Proteins were identified with a chemiluminescence western blotting detection system (Bio-Rad). Three Western blot experimental replicates were conducted for each molecular assay.

### RNAi and cell transfection

SiRNAs (si-circ#1, si-circ#2, si-circ#3) and siNC (RiboBio, Guangzhou, China) designed for the back-splicing sites of circNDST1 were used to specifically knock down circNDST1 without affecting the linear NDST1 molecular expression. The siRNA sequences were as follows:

si-circ#1 ACCCGCTCTGGCAGGTTCT;

si-circ#2 GCTCTGGCAGGTTCTCCCA;

si-circ#3 TCTGGCAGGTTCTCCCACG.

The siRNA sequences used to knock down CSNK2A1 were as follows:

si-CSNK2A1#1: GTCAGCAGCGCCAATATGA;

si-CSNK2A1#2: GGTGAGGATAGCCAAGGTT;

si-CSNK2A1#3: GTTTGGATATGTGGAGTTT;

Transfections were performed with Liposome 3000 (Invitrogen, Carlsbad, CA, USA, cat. NO: CA92008) according to the manufacturer's protocol. Stably transfected TPC1 cell lines used to knock down circNDST1 were constructed with a lentivirus purchased from Genechem (Shanghai, China) and were screened with puromycin (Invitrogen, Carlsbad, CA, USA, cat. NO: J67236.XF).

### EdU incorporation assay

The EdU assay was conducted using a Cell-Light EdU DNA Cell Proliferation Kit (RiboBio, Guangzhou, China, cat. NO: C10310-1). TPC1 cells were incubated with 50 mM EdU for 2 h, fixed in 4% paraformaldehyde, and stained with an Apollo Dye Solution, and their nuclei were identified using Hoechst 33,342. Proliferation-positive cells were photographed and counted under a fluorescence microscope.

### CCK8, colony formation assay, transwell migration and invasion assay, and wound healing assay

CCK8 (Bimake, cat. NO: B34304), colony formation assay, transwell migration and invasion assay, and wound healing assay were performed as previously reported [13, 14].

### Biotin-coupled probe RNA pull-down assay and mass spectrometry

A biotinylated circNDST1 probe (5´ CTGCCGTGGGAGAACCTGCCAGAGCGGGTC 3´) and a control probe (5´ GACCCGCTGCTGGCAGGTTCTCCCACGGCAG 3´) synthesized by Sangon (Shanghai, China) were used to pull down the protein bound to circRNA. Approximately 1 × 10^7^ TPC1 cells were lysed and incubated with biotin-labeled probes, and the biotin-coupled RNA protein complex was pulled down using streptavidin affinity-coated magnetic beads (MedChemExpress, cat. NO: HY-K0208), which were then scrutinized with mass spectrometry (SpecAllly Life Sciences Co. Wuhan, China) to identify the protein bound to circNDST1.

### RNA immunoprecipitation (RIP)

The RIP assay was carried out using a Magna RIP RNA Binding Protein Immunoprecipitation Kit (Millipore, USA, cat. NO: 17-700) according to the manufacturer’s instructions.

### Transcriptome RNA sequencing

Transfected TPC1 cells were lysed with Trizol, and the changes in the transcript levels of TPC1 cells in the lysate were determined by Haplox (Jiangxi, China) after the knockdown of circNDST1. KEGG pathway aggregation analysis and transcriptome volcano mapping were performed using Sangerbox tools, a free online platform for data analysis (http://vip.sangerbox.com/).

### Protein immunoprecipitation

Approximately 1 × 10^5^ TPC1 cells were lysed with an IP lysis solution (Beyotime, Shanghai, China, cat. NO: P0013) and incubated separately overnight with 5 μg anti-CSNK2A1 antibody and IgG (Proteintech). The antibody-protein complex was then pulled down using protein A/G magnetic beads (MedChenExpress,cat.NO:HY-K0202), and protein molecules bound to the target protein were spotted using western blot.

### Nude mice tumorigenesis assay

Four-week-old female BALB/C nude mice were selected for the xenograft experiment and kept under specific pathogen-free conditions. The experiment was approved by the Institutional Animal Care and Use Committee of Huazhong University of Science and Technology (Ethical number: S2841).

Overall, 10 nude mice were used for the xenograft experiments: 5 mice in the experimental group and 5 in the control group. Approximately 1 × 10^9^ TPC1 stably transfected cells mixed with a matrix gel (Corning, USA, cat. NO: 354234) were injected subcutaneously into nude mice. Tumor volumes measurements (length × width^2^/2) started 2 weeks after inoculation. The nude mice were executed 6 weeks after inoculation with cells, the tumors were removed, and the tumor volume and weight were measured.

### Statistical analysis

Statistical analyses were conducted primarily using SPSS 21.0 (IBM, SPSS, Chicago, IL, USA) and GraphPad Prism 6.0 (GraphPad Software Inc., CA, USA). Differences between groups were analyzed using Student’s *t*-test and one-way ANOVA.

## Results

### Increased circNDST1 expression in PTC and characteristics of circNDST1

**Fig. 1:**
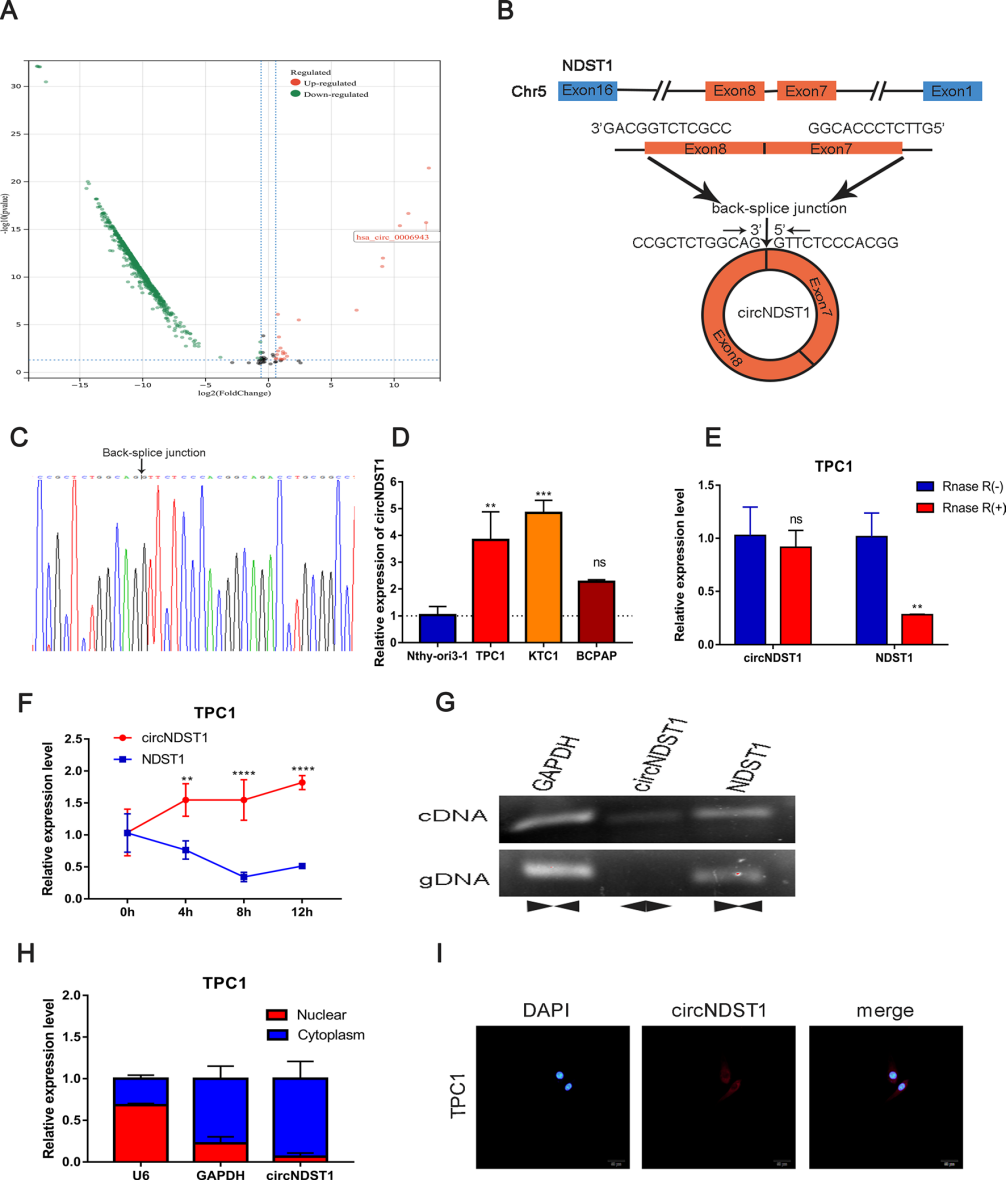
The expression and characteristics of hsa_circ_0006943.** A** Volcano map of circRNA high-throughput sequencing of 3 cases of PTC with extensive lymph node metastasis in the neck versus 3 cases of PTC without extensive lymph node metastasis. **B** Schematic diagram of the formation structure of circNDST1. **C** sanger sequencing to identify circNDST1 back-splicing site. **D** The expression of circNDST1 in normal thyroid epithelial cells and PTC cell lines. **E** Rnase R resistance of circNDST1 and its linear molecule NDST1. **F** After treatment of TPC1 cells with actinomycin D, the levels of circNDST1 and linear molecule NDST1 were detected by RT-qPCR at different treatment times. **G** PCR amplification of cDNA and gDNA in GAPDH, circNDST1,NDST1 and subsequent agarose gel electrophoresis. **H** Nucleoplasmic separation was used to detect circNDST1 localization, where U6 was used as a cytosolic reference and GAPDH as a cytoplasmic reference. **I** FISH detection of the localization of circNDST1 (scale bar: 50 μm). *P < 0.05, **P < 0.01, ***P < 0.001. Data are shown as SD ± mean

PTC tissues with and without extensive lymph node metastasis were high-throughput sequenced. Patients’ clinicopathological information and sequencing results are shown in the attached table. Tissues with extensive lymph node metastasis were screened for significantly up-regulated circRNAs. The sequenced molecules were plotted as volcanoes, with 17 circRNAs considerably up-regulated (Fig. 1A). The first five significantly up-regulated circRNAs were selected for pre-experimentation.

For the back-splicing site of circRNAs, specific divergent primers were designed for assessing the expression of circRNAs in the cell lines. The amplified products of RT-qPCR were then subjected to sanger sequencing to verify the specific presence of the back-splicing site. Ultimately, based on the PCR and sanger sequencing results, hsa_circ_0006943 (circNDST1) was chosen as the target molecule for this investigation.

CircNDST1 was formed by the back-splicing of exon 7 and exon 8 of N-deacetylase/N-sulfotransferase 1 (NDST1) (Fig. 1B), with sanger sequencing establishing the presence of the back-splicing site (Fig. 1C). CircNDST1 expression was significantly higher in PTC cell lines (TPC1, KTC1, BCPAP) than in normal thyroid epithelial cells (Nthy-ori3-1) (Fig. 1D). While RNase R degraded the linear NDST1 molecule, it did not affect circNDST1 expression, indicating that circNDST1 is resistant to RNase R digestion (Fig. 1E). The circular structure of circRNA rendered circNDST1 more stable and less susceptible to degradation than the linear NDST1 molecule after treatment with actinomycin D (Fig. 1F).

Specific divergent primers for the back-splicing site of circNDST1 were designed, and agarose gel electrophoresis revealed no presence of circNDST1 in the gDNA (Fig. 1G). Nucleoplasmic separation experiments (Fig. 1H) and FISH assays (Fig. 1I) established that circNDST1 was mainly localized in the cytoplasm.

### CircNDST1 promotes the proliferation, migration, invasion, and the EMT of PTC* in vitro* and* in vivo*

**Fig. 2:**
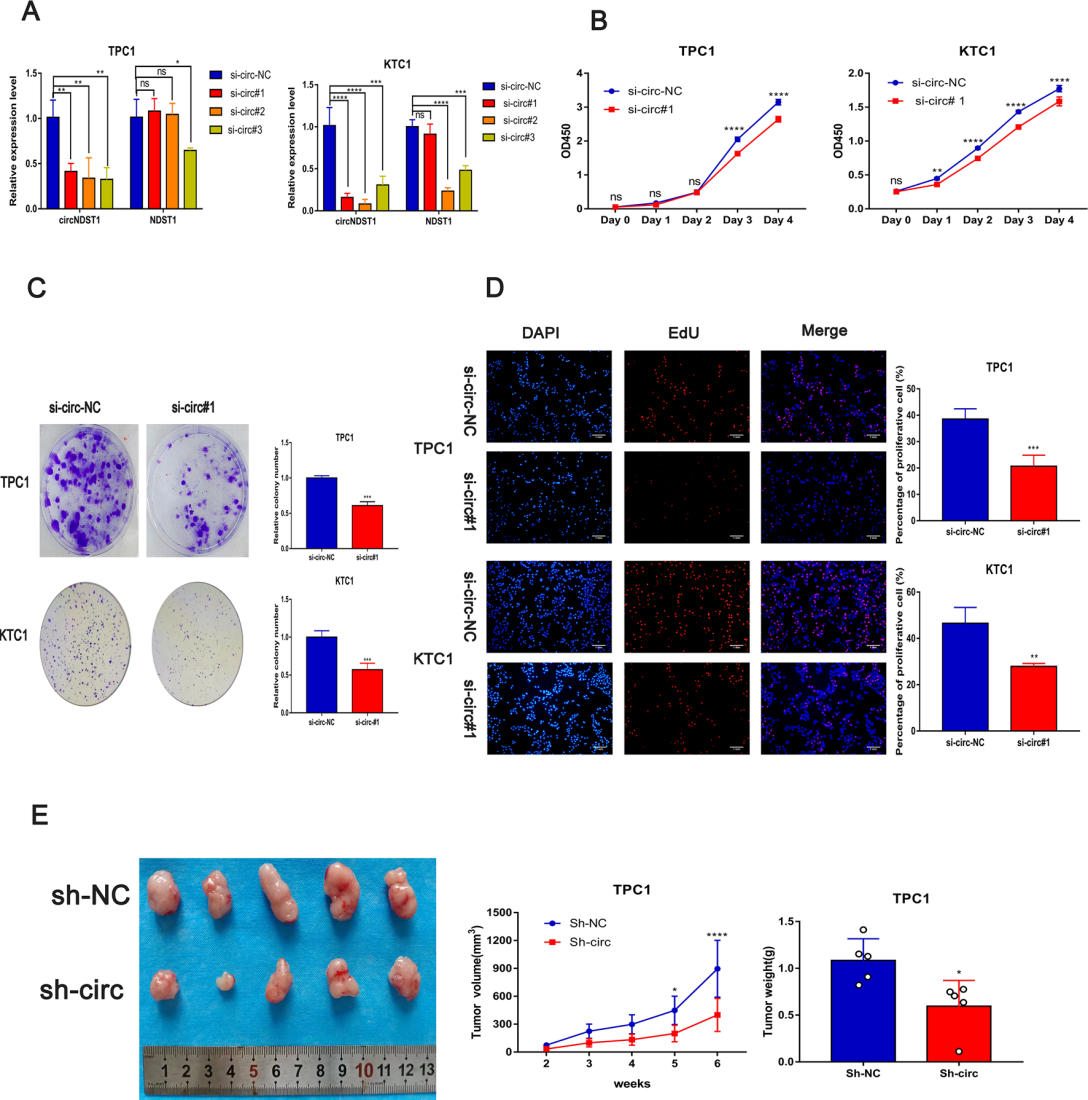
CircNDST1 promotes thyroid cancer proliferation in vitro and in vivo.** A** Knockdown of circNDST1 using siRNA in TPC1 and KTC1 cell lines,RT-qPCR to detect circNDST1 and NDST1 expression. **B** The growth curve of the cells was measured by CCK8 assay. **C** The ability of cells to proliferate was assessed using clony formation assays. **D** EdU assay to assess cell proliferation capacity (scale bar: 1 mm). **E** TPC1 cells with stably knocked down circNDST1 were used for tumorigenesis experiments in nude mice, and tumor volume and weight were measured. *P < 0.05, **P < 0.01, ***P < 0.001, ****P < 0.0001. Data are shown as SD ± mean

TPC1 and KTC1 cells were transfected with specific siRNAs designed for the circNDST1 back-splicing site. Because circNDST1 is formed by the cyclization of exons 7 and 8 of the linear NDST1 molecule, siRNA, when used to knock down circNDST1, can easily produce off-target effects to incorrectly knock down the linear NDST1 molecule. Therefore, for the sake of experimental precision, si-circ#1 was selected for subsequent functional experiments to avoid disrupting the expression of the linear NDST1 molecule (Fig. 2A). CCK8 (Fig. 2B) and plate cloning assays (Fig. 2C) revealed that circNDST1 enhanced the proliferation of TPC1 and KTC1 cells in vitro. In the EdU experiments, TPC1 and KTC1 cells had reduced proportions of cells in the proliferative state after the knockdown of circNDST1 (Fig. 2D).

To scrutinize the influence of circNDST1 on PTC in vivo, nude mice were subjected to tumorigenic experiments using TPC1 cells with a stable knockdown of circNDST1. The results showed reduced tumor volume and weight after the knockdown of cirNDST1, confirming that circNDST1 promotes the proliferation of thyroid cancer cells in vivo (Fig. 2E). CCK8, plate clone, and EdU experimental results after knocking down circNDST1 using si-circ#2 are shown in supplementary Fig. 2.

**Fig. 3:**
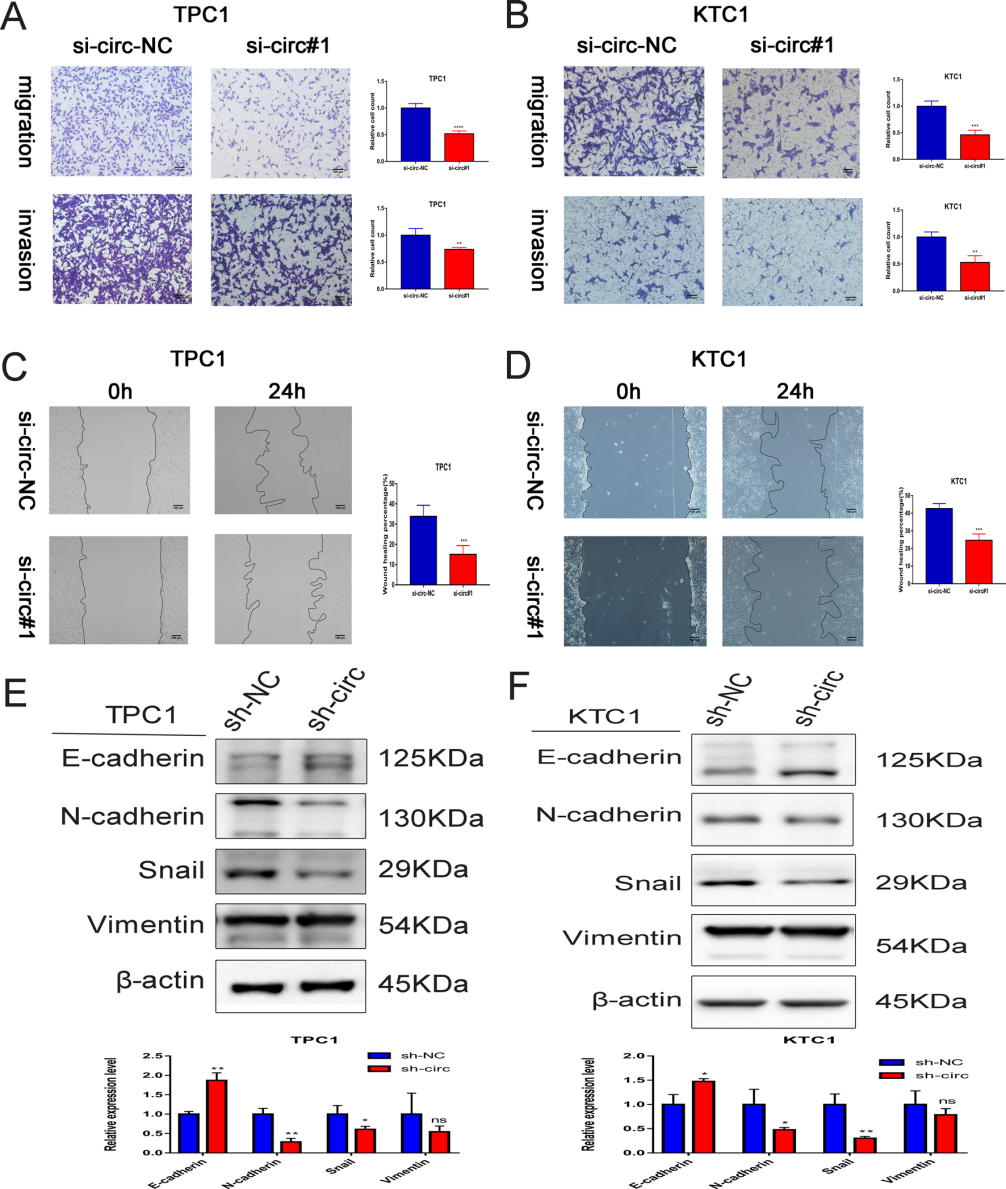
CircNDST1 promotes thyroid cancer migration and invasion through EMT. **A** Effect of circNDST1 on the migratory invasion ability of TPC1 cells assessed by Transwell assay(scale bar:100 μm). **B** Effect of circNDST1 on the migratory invasion ability of KTC1 cells assessed by Transwell assay(scale bar:100 μm). **C** Effect of circNDST1 on the migratory capacity of TPC1 cells assessed by wound healing assay(scale bar:100 μm). **D** Effect of circNDST1 on the migratory capacity of KTC1 cells assessed by wound healing assay(scale bar:100 μm). **E** Effect of circNDST1 knockdown on EMT essential protein expression in TPC1 cells detected by Western blot. **F** Effect of circNDST1 knockdown on EMT essential protein expression in KTC1 cells detected by Western blot. *P < 0.05, **P < 0.01, ***P < 0.001. Data are shown as SD ± mean

As per the Transwell assay, the migration and invasion abilities of TPC1 and KTC1 cells lessened after the knockdown of circNDST1 (Fig. 3A, B). The scratch wound healing experiment also established that knocking down circNDST1 diminished the migratory capacity of TPC1 and KTC1 (Fig. 3C, D). Western blot uncovered increased E-cadherin expression and decreased N-cadherin, Vimentin, and Snail expression after the stable knockdown of circNDST1 (Fig. 3E, F), indicating that circNDST1 stimulates EMT in PTC. Transwell and scratch wound healing experimental findings after circNDST1 knockdown using si-circ#2 are shown in supplementary Fig. 3.

### CircNDST1 affects CCND1 expression through the activation of the PI3K–Akt pathway and promotes PTC progression

**Fig. 4:**
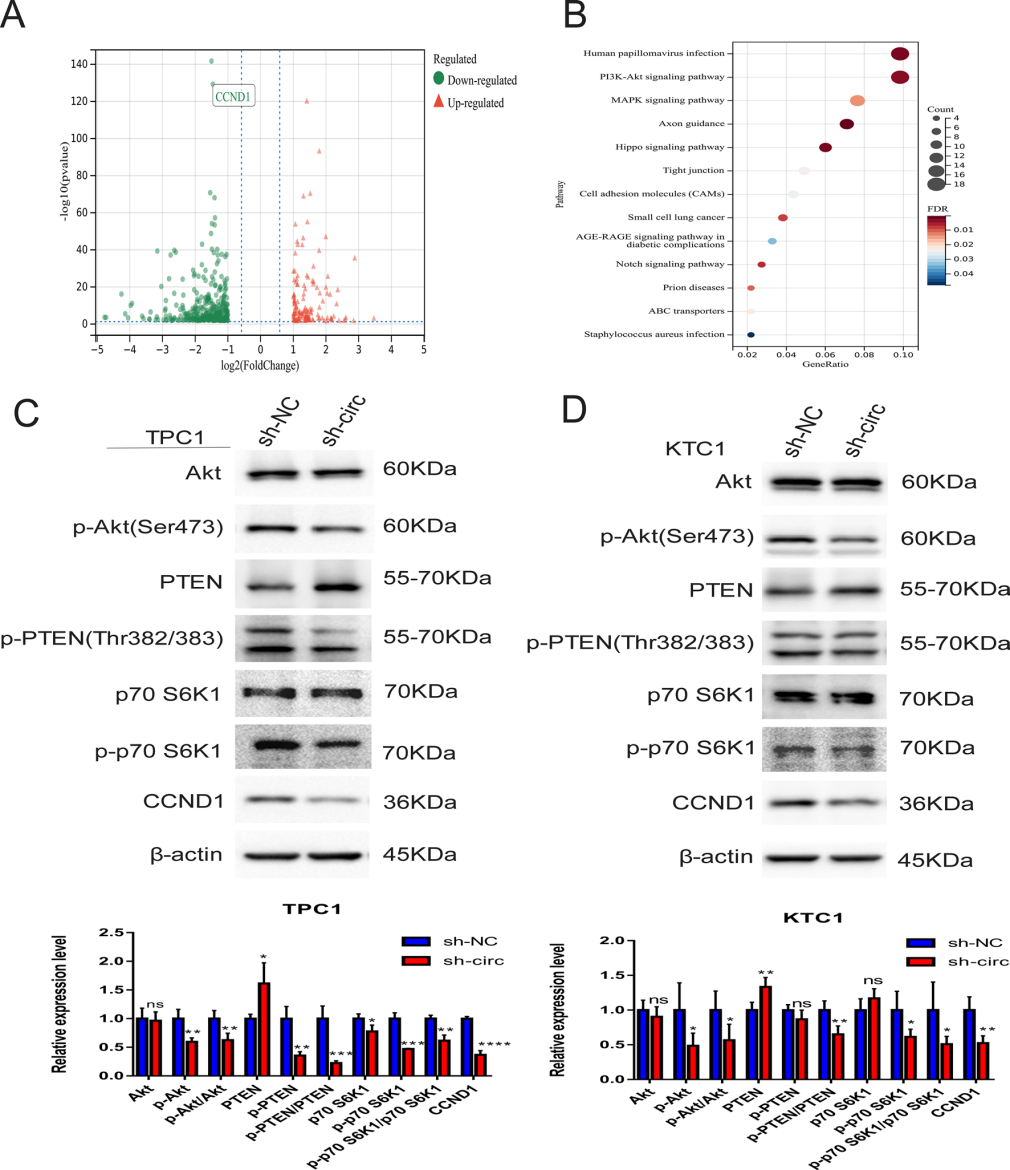
CircNDST1 promotes CCND1 expression via PI3K–Akt pathway and promotes thyroid cancer progression.** A** Knockdown of circNDST1 using si-circ#1 in TPC1 cells followed by transcriptome sequencing to map the differential genes (*P* < 0.05, log_2_FC > 1) into volcanoe. **B** KEGG clustering analysis of transcriptome sequencing, plotting bubble plot of differential pathways meeting *P* < 0.05. **C** Effect of circNDST1 on the expression of essential proteins of PI3K–Ak pathway in TPC1 cells by Western blot. **D** Effect of circNDST1 on the expression of essential proteins of PI3K–Ak pathway in KTC1 cells by Western blot. *P < 0.05, **P < 0.01, ***P < 0.001, ****P < 0.0001. Data are shown as SD ± mean

To identify the mechanism through which circNDST1 affects thyroid carcinogenesis, transcriptome sequencing was used to examine changes in TPC1 cells at the RNA level after circNDST1 knockdown with si-circ#1. Knocking down circNDST1 resulted in the alteration of 475 genes (log2FC > 1, *P* < 0.05) at the mRNA level. Using the online tool, Sangerbox, these genes were mapped to volcanoes, where the expression of cyclin D1 (CCND1) decreased after circNDST1 knockdown (Fig. 4A). The pathways from the KEGG pathway aggregation analysis that met the *P* < 0.05 condition were plotted as bubble plots, and this revealed that the PI3K–Akt pathway had the highest number of altered genes (Fig. 4B). The transcriptome sequencing results after circNDST1 was knocked down using si-circ#2 are shown in Supplementary Fig. 4.

The PI3K–Akt signaling pathway is an essential oncogenic driver of thyroid cancer [15, 16]. The tensin homolog deleted on chromosome 10 (PTEN) is a tumor suppressor that negatively regulates the PI3K–Akt pathway through dephosphorylation [17, 18]. According to our Western blot analyses, knocking down circNDST1 saw the phosphorylation levels of Akt, PTEN, and p70 S6K1 decrease, PTEN protein expression increase, and CCND1 expression decline (Fig. 4C, D). This suggests that knocking down circNDST1 inhibits the activation of the PI3K–Akt pathway in thyroid cancer.

### CircNDST1 promotes CSNK2A1 binding to Akt by interacting with CSNK2A1

**Fig. 5:**
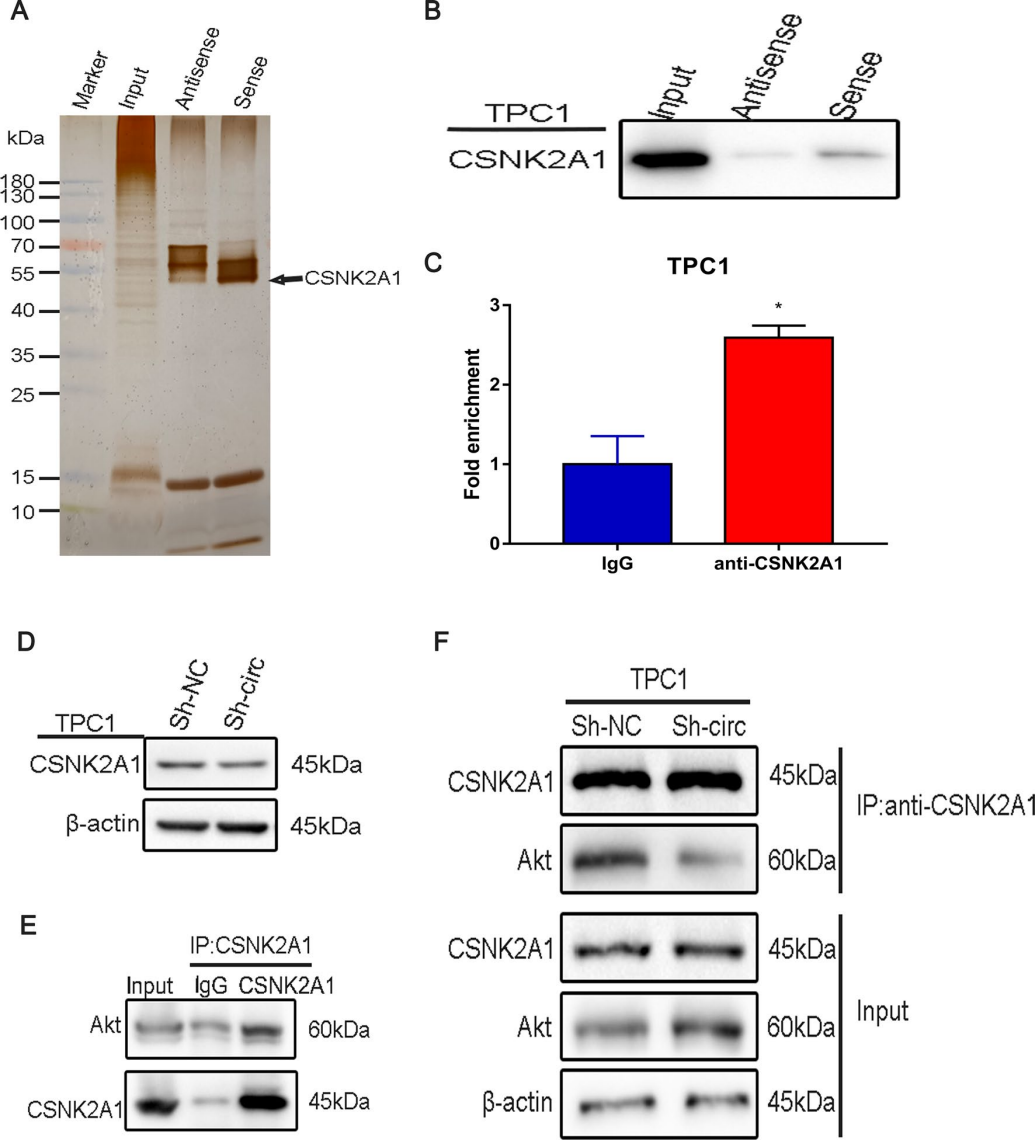
CircNDST1 promotes CSNK2A1 binding to Akt by interacting with CSNK2A1. **A** The specific biotin-labeled circNDST1 probe was added to the TPC1 lysate, and the precipitated proteins were resolved by SDS-PAGE, followed by silver staining. Next, the differential bands in the lane of the circNDST1 sense probe were identified by mass spectrometry (MS). The antisense of circNDST1 was used as a control. As shown in the figure, CSNK2A1 was identified. **B** RNA pull-down assay with circNDST1 probe and antisense probe and verification of CSNK2A1-specific presence in the experimental group. **C** RIP experiment was performed with anti-CSNK2A1 antibody, and the abundance of circNDST1 in RNA precipitates was detected by RT-qPCR. IgG was used as a control group. *P < 0.05 **D** Effect of knockdown of circNDST1 on CSNK2A1 detected by Western blot. **e** Protein immunoprecipitation with anti-CSNK2A1 antibodies and verification of CSNK2A1 binding to Akt by Western blot. **f** TPC1 cells with knockdown circNDST1 were incubated with anti-CSNK2A1 antibody for protein immunoprecipitation and Western blot to detect changes in precipitated Akt

Using FISH and nucleoplasmic separation experiments, we determined that circNDST1 was localized mainly in the cytoplasm. We speculated that circNDST1 possibly acts by binding to a specific protein and confirmed this theory by establishing via the RNA pull-down method of biotin-labeled probes, silver staining (Fig. 5A), mass spectrometry, and subsequent western blot validation that circNDST1 functions by binding to CSNK2A1 (Fig. 5B). RIP experiments corroborated the binding of CSNK2A1 to circNDST1 (Fig. 5C). Knocking down circNDST1 did not cause a significant change in the protein expression of CSNK2A1 (Fig. 5D).

We next hypothesized that circNDST1 probably promotes the action of CSNK2A1 through the adsorption of CSNK2A1. IP experiments confirmed the binding of CSNK2A1 to Akt in TPC1 cells (Fig. 5E). Knocking down circNDST1 saw the Akt bound to CSNK2A1 pulled down by the anti-CSNK2A1 antibody, with the pulled down Akt reduced (Fig. 5F), demonstrating that circNDST1 boosts CSNK2A1 binding to Akt.

### CSNK2A1 enhances PTC progression

**Fig. 6:**
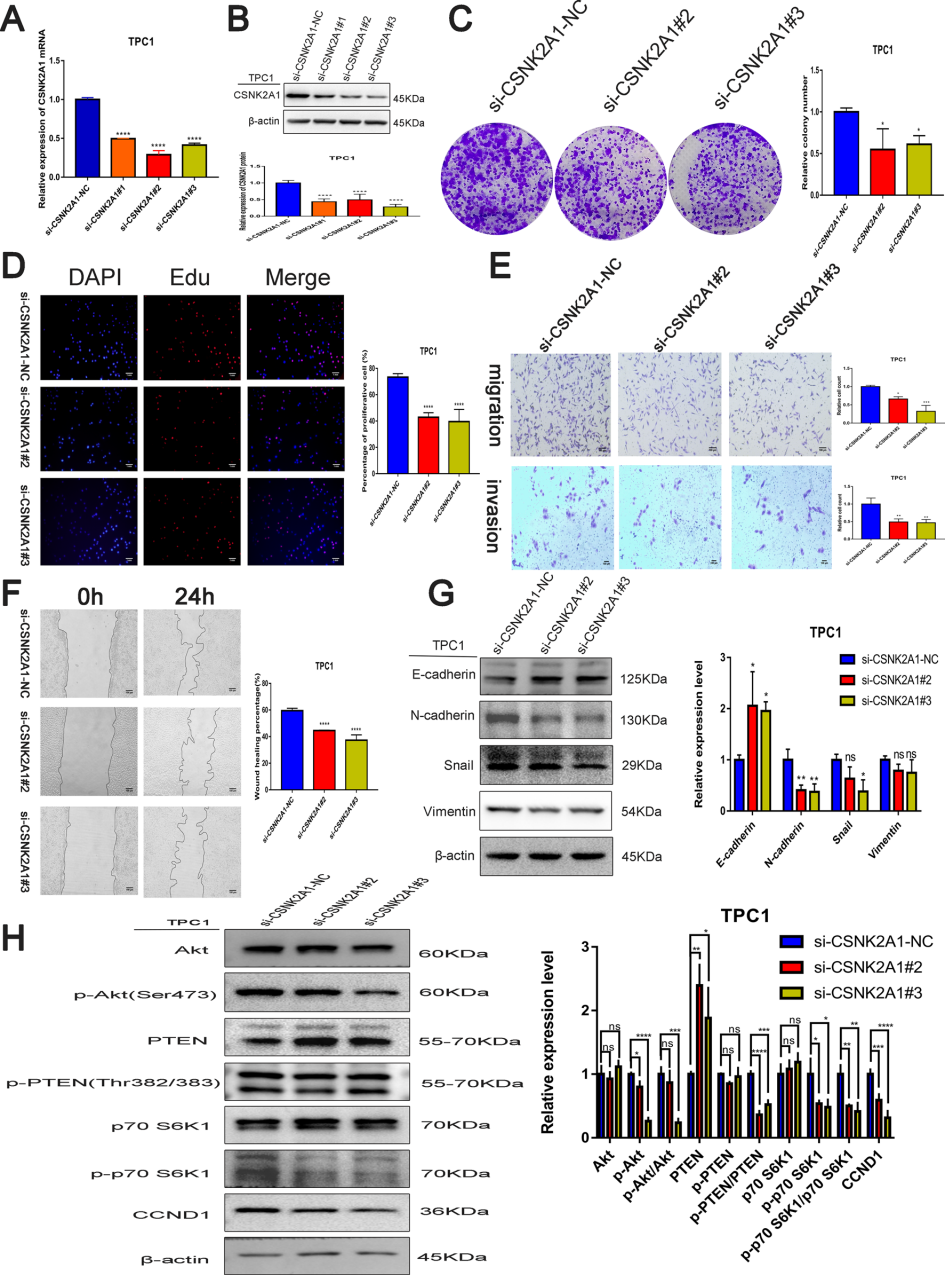
CSNK2A1 promotes the proliferation, migration, invasion and EMT of thyroid cancer cells through PI3K–Akt pathway.** A**, **B** Knockdown of CSNK2A1 by siRNA, RT-qPCR at mRNA level and western blot at protein level. Effect of CSNK2A1 on the proliferative capacity of thyroid cancer cell lines assessed by clony formation assay (**C**) and EdU assay(**D** scale bar:1 mm). Effect of CSNK2A1 on migration and invasive ability of thyroid cancer cell lines assessed by transwell (**E** scale bar:100 μm) and scratch assays (**F** scale bar:100 μm). **G** Effect of circNDST1 on the expression of essential proteins of EMT process in TPC1 cells by Western blot. **H** Effect of circNDST1 on the expression of essential proteins of PI3K–Akt pathway in TPC1 cells by Western blot. *P < 0.05, **P < 0.01, ***P < 0.001, ****P < 0.0001. Data are shown as SD ± mean

CSNK2A1 was knocked down in TPC1 cells using siRNA, and the knockdown effect was examined at the mRNA (Fig. 6A) and protein (Fig. 6B) levels, resulting in the selection of si-CSNK2A1#2 and si-CSNK2A1#3 for subsequent experiments. Per the colony formation assay, the knockdown of CSNK2A1 inhibited the proliferative capacity of TPC1 cells (Fig. 6C). EdU assays revealed that CSNK2A1 knockdown reduced the proportion of TPC1 cells in the proliferative state (Fig. 6D), and transwell (Fig. 6E) and scratch wound healing (Fig. 6F) assays established that knocking down CSNK2A1 subdued the migration and invasion abilities of TPC1 cells. According to findings from the Western blot analysis, knocking down CSNK2A1 repressed EMT and the PI3K–Akt pathway activity, as shown by increased E-cadherin and PTEN and decreased N-cadherin, Vimentin, Snail, and p-Akt, p-PTEN, p-p70 S6K1, and CCND1 (Fig. 6G, H).

## Discussion

The rate of thyroid cancer incidence is increasing year after year, the vast majority of which is PTC [19, 20]. Despite the good prognosis of PTC, cervical lymph node metastasis remains a determining factor for surgical choice and distant metastasis. Therefore, outlining the underlying molecular mechanisms that lead to cervical lymph node metastasis in PTC is critical to the development of new anti-tumor strategies.

circRNA affects tumor development in a variety of tumors, including breast cancer [21], colon cancer [22], renal cell carcinoma [23], and bladder cancer [24], through various mechanisms influencing tumor cell proliferation, migration, invasion, DNA damage repair, epigenetic modifications, and drug resistance. In this investigation, three PTCs with extensive neck lymph node metastasis and three PTCs without extensive lymph node metastasis were high-throughput sequenced for key circRNAs involved in neck lymph node metastasis in PTCs. Hsa_circ_0006943 (circNDST1) was identified as the most decisive target. CCK8, plate cloning, EdU, transwell, and scratch assays, as well as tumor formation in nude mice, revealed that circNDST1 promoted thyroid cancer proliferation, migration, and invasion.

Our study provides a rigorous approach to studying the function of circRNAs. CircRNAs are essentially formed by the exon and intron splicing and cyclization of their linear molecules, suggesting that when knocking down circRNAs, siRNAs can easily produce off-target effects and incorrectly knock down linear molecules. Because subsequent meticulous functional studies did not confirm that the anticancer effect is produced by circRNA alone, si-circ#1, which did not upset the linear NDST1 molecule, was selected for functional experiments when knocking down circNDST1. However, we did not expand the clinical sample size to fully validate the correlation between circNDST1 and thyroid cancer lymph node metastasis, and that is a dent in our research.

EMT confers metastatic properties to cancer cells by enhancing tumor cell migration, invasion, and resistance to apoptosis. This process is, therefore, considered a marker of carcinogenesis [25, 26]. Additionally, the activation of the PI3K–Akt signaling pathway enhances tumor cell invasion and oncogenic gene expression via the phosphorylation of Akt [27]. PTEN inhibits tumor invasion by suppressing the PI3K–Akt signaling pathway and counteracting its cascade response [28]. In the present study, we demonstrated that inhibiting circNDST1 expression decreased the phosphorylation levels of Akt, p70 S6K1, and PTEN (a tumor suppressor) and increased the expression of PTEN, thus preventing the activation of the PI3K–Akt pathway. CircNDST1 also affected the expression of E-cadherin, N-cadherin, Vimentin, Snail, and other key molecules of EMT. CircNDST1 is a pro-oncogenic molecule of the PI3K–Akt signaling pathway and EMT.

CSNK2A1 has been shown to affect tumor recurrence, metastasis, and prognosis through the phosphorylation of various substrates, making it a key target for anti-tumor therapy [6, 29–32]. CSNK2A1 encodes the catalytic subunit of protein kinase CK2 and enhances Akt activity by phosphorylating Akt. It also phosphorylates PTEN, which reduces PTEN stability and alters PTEN localization in cells, thereby activating the PI3K–Akt pathway to promote tumor cell growth, adhesion, and migration [5, 11]. In the present study, we queried the binding of circNDST1 to CSNK2A1 via biotin-coupled probe pull-down and RIP assay and also confirmed CSNK2A1’s pro-carcinogenic role in thyroid cancer through a series of functional experiments. Immunoprecipitation and western blot revealed that CSNK2A1 activates the PI3K–Akt pathway by interacting with Akt and phosphorylating Akt. Our subsequent speculation that circNDST1 activates the PI3K–Akt pathway by reinforcing the binding of CSNK2A1 to Akt was validated by IP experiments after the knockdown of circNDST1. CSNK2A1, a kinase, becomes a hub between the circNDST1 and PI3K–Akt pathways. This mechanism also gives rise to new concepts for circRNA mechanistic studies, bringing CSNK2A1 to the fore as a new target for thyroid cancer.

In the present study, we demonstrated that has_circ_0006943 promotes the development of thyroid cancer by binding to CSNK2A1, facilitating the binding of CSNK2A1 to Akt, and subsequently activating PI3A–Akt and EMT. To sum up, by using circRNA high-throughput sequencing and functional validation, our investigation has demonstrated the potential of circNDST1 as a prognostic biomarker for thyroid cancer. In particular, the cyclization properties of circNDST1 and its stability offer the possibility of circNDST1 as a stable serum marker for lymph node metastasis prediction in thyroid cancer. Mechanistically, we established through rigorous experiments that CSNK2A1, a kinase, acts as an important bridge between circNDST1 and the PI3K–Akt pathway. This also renders CSNK2A1 a feasible new biomarker and future therapeutic target for thyroid cancer, paving the way for novel drug interventions in thyroid cancer.

## Supplementary Information

Below is the link to the electronic supplementary material.

Supplementary file1 (XLSX 102 KB)

Supplementary file2 (XLSX 10 KB)

Fig. 2 supplementary CircNDST1 promotes thyroid cancer proliferation by using another siRNA to knockdown circNDST1. a The growth curve of the cells was measured by CCK8 assay. b The ability of cells to proliferate was assessed using clony formation assays. c EdU assay to assess cell proliferation capacity(scale bar:1mm).*P<0.05,**P<0.01,***P<0.001,****P<0.0001. Data are shown as SD ± mean

Fig. 3 supplementary CircNDST1 promotes thyroid cancer migration and invasion using another siRNA to knockdown circNDST1. a Effect of circNDST1 on the migratory invasion ability of TPC1 cells assessed by Transwell assay(scale bar:100μm). b Effect of circNDST1 on the migratory invasion ability of KTC1 cells assessed by Transwell assay(scale bar:100μm). c Effect of circNDST1 on the migratory capacity of TPC1 cells assessed by wound healing assay(scale bar:100μm). d Effect of circNDST1 on the migratory capacity of KTC1 cells assessed by wound healing assay(scale bar:100μm). *P<0.05,**P<0.01,***P<0.001. Data are shown as SD ± mean

Fig. 4 supplementary CircNDST1 promotes thyroid cancer progression via PI3K–Akt pathway. a Knockdown of circNDST1 using si-circ#2 in TPC1 cells followed by transcriptome sequencing to map the differential genes into volcanoe. b KEGG clustering analysis of transcriptome sequencing, plotting bubble plot of differential pathway

Supplementary file6 (XLS 45 KB)

Supplementary file7 (XLS 1472 KB)

Supplementary file8 (XLS 1060 KB)
