# Supplementary figures and images for: CircNDST1 promotes papillary thyroid cancer progression via its interaction with CSNK2A1 to activate the PI3K–Akt pathway and epithelial–mesenchymal transition
Source: J Endocrinol Invest. 2022 Oct 28;46(3):545–57. doi: 10.1007/s40618-022-01928-x (PMC9938055; doi:10.1007/s40618-022-01928-x)

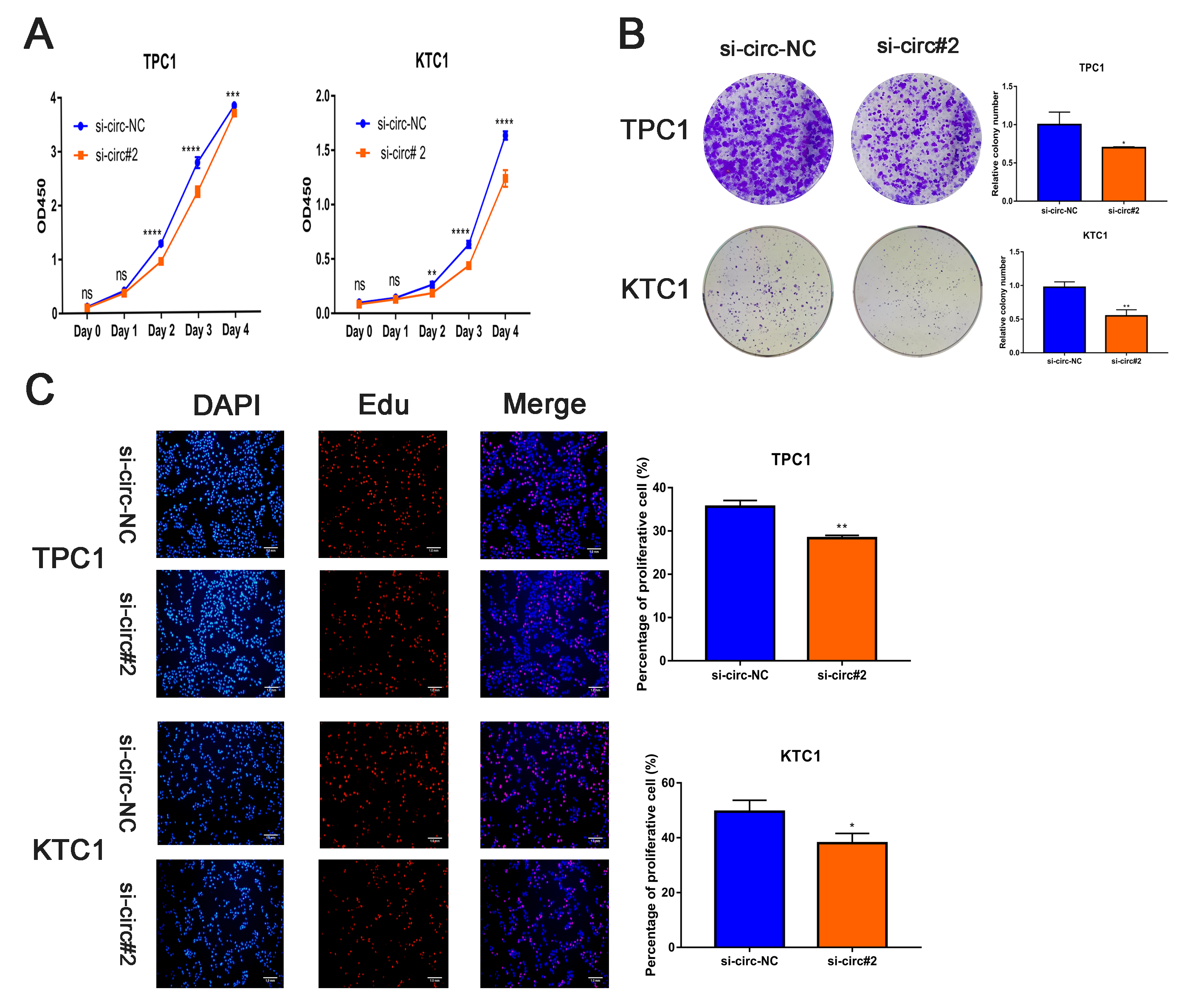

Supplement: Supplementary file 3 — Fig. 2 supplementary CircNDST1 promotes thyroid cancer proliferation by using another siRNA to knockdown circNDST1. a The growth curve of the cells was measured by CCK8 assay. b The ability of cells to proliferate was assessed using clony formation assays. c EdU assay to assess cell proliferation capacity(scale bar:1mm).*P<0.05,**P<0.01,***P<0.001,****P<0.0001. Data are shown as SD ± mean [file 40618_2022_1928_MOESM3_ESM.tif]

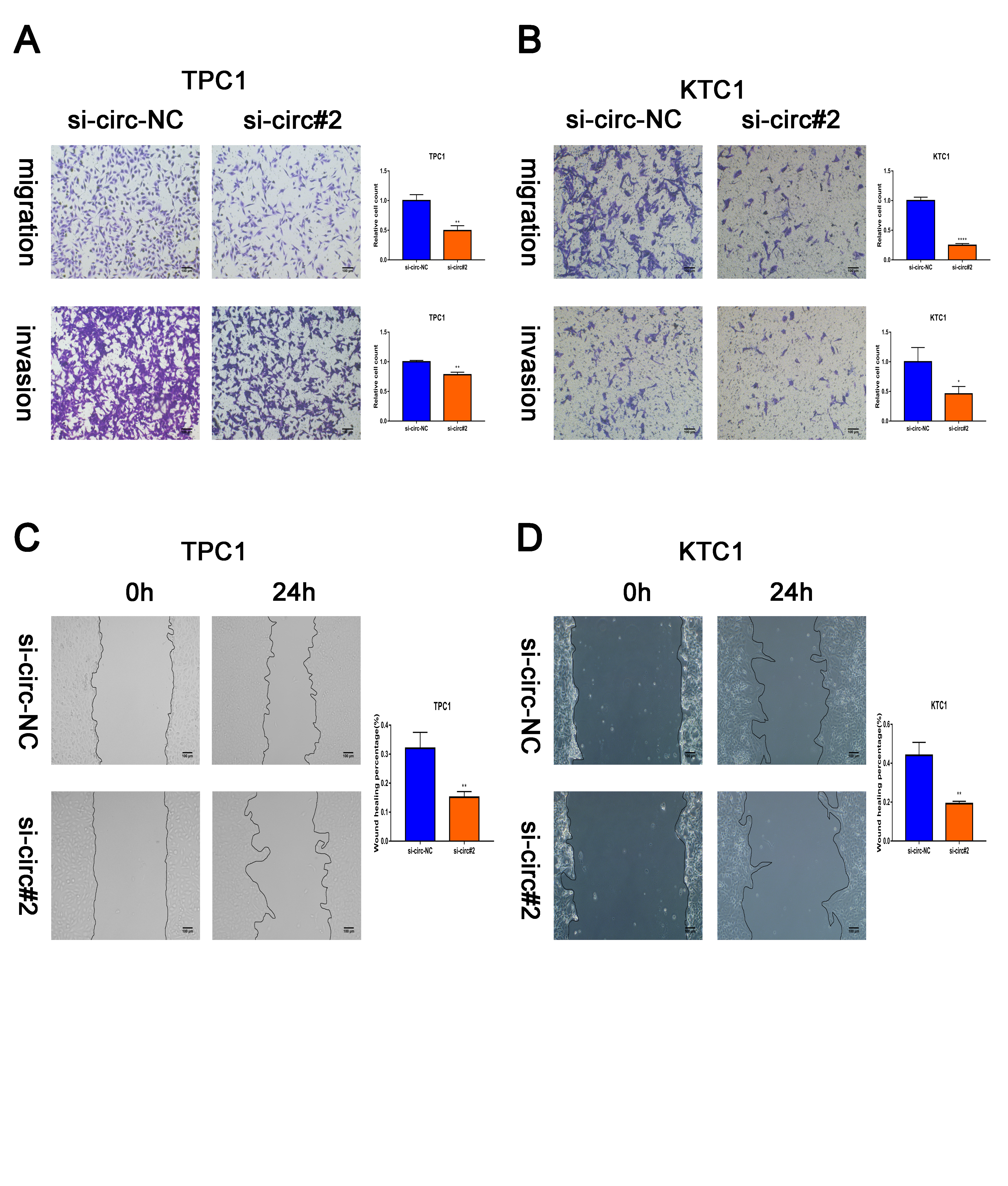

Supplement: Supplementary file 4 — Fig. 3 supplementary CircNDST1 promotes thyroid cancer migration and invasion using another siRNA to knockdown circNDST1. a Effect of circNDST1 on the migratory invasion ability of TPC1 cells assessed by Transwell assay(scale bar:100μm). b Effect of circNDST1 on the migratory invasion ability of KTC1 cells assessed by Transwell assay(scale bar:100μm). c Effect of circNDST1 on the migratory capacity of TPC1 cells assessed by wound healing assay(scale bar:100μm). d Effect of circNDST1 on the migratory capacity of KTC1 cells assessed by wound healing assay(scale bar:100μm). *P<0.05,**P<0.01,***P<0.001. Data are shown as SD ± mean [file 40618_2022_1928_MOESM4_ESM.tif]

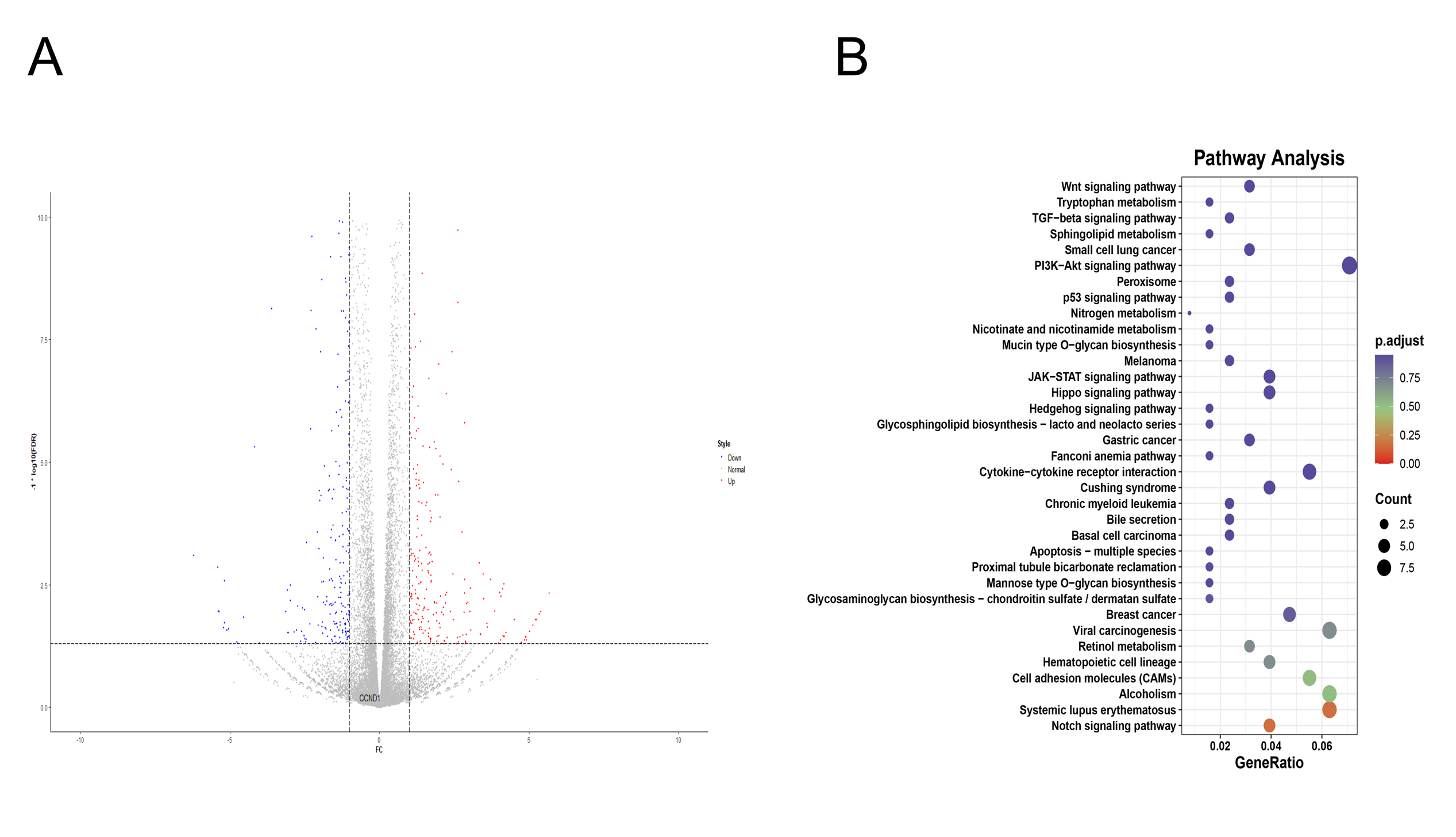

Supplement: Supplementary file 5 — Fig. 4 supplementary CircNDST1 promotes thyroid cancer progression via PI3K–Akt pathway. a Knockdown of circNDST1 using si-circ#2 in TPC1 cells followed by transcriptome sequencing to map the differential genes into volcanoe. b KEGG clustering analysis of transcriptome sequencing, plotting bubble plot of differential pathway [file 40618_2022_1928_MOESM5_ESM.tif]
